# Magnetic self-assembly of 3D multicellular microscaffolds: A biomimetic brain tumor-on-a-chip for drug delivery and selectivity testing

**DOI:** 10.1063/5.0155037

**Published:** 2023-07-24

**Authors:** Attilio Marino, Matteo Battaglini, Alessio Carmignani, Francesca Pignatelli, Daniele De Pasquale, Omar Tricinci, Gianni Ciofani

**Affiliations:** 1 Istituto Italiano di Tecnologia, Smart Bio-Interfaces, Viale Rinaldo Piaggio 34, 56025 Pontedera, Italy; 2 Scuola Superiore Sant’Anna, The BioRobotics Institute, Viale Rinaldo Piaggio 34, 56025 Pontedera, Italy

## Abstract

In recent years, the need for highly predictive brain cancer models to test new anticancer compounds and experimental therapeutic approaches has significantly increased. Realistic *in vitro* brain tumor-on-a-chip platforms would allow a more accurate selection of valid candidate drugs and nanomedicines, therefore alleviating the economic and ethical issues of unsuccessful studies *in vivo*. Here, we present a multi-functional self-assembled brain tumor-on-a-chip model characterized by 3D glioma cultures interfaced both to nonmalignant brain cells of the peritumoral niche and to a 3D-real-scale blood–brain barrier (BBB) microfluidic system. This platform allowed us to screen multiple features, such as BBB crossing capabilities, apoptotic efficacy against GBM cells, and side effects on nonmalignant brain cells of a promising anticancer drug, nutlin-3a, which is fundamental for the treatment of brain cancer.

## Introduction

Brain and central nervous system (CNS) cancers represent a major public health burden, with a progressive increase in incidence, deaths, and disability-adjusted life years (DALY).^1,2^ Glioblastoma multiforme (GBM) is a highly malignant, aggressive, and generally deadly brain cancer. GBM shows frequent and rapid recurrence, resulting in only 4% 5-year survival despite gold-standard treatments (i.e., temozolomide-based chemotherapy and radiation).^3,4^ The cell clusters infiltrated in the peritumoral niche that cannot be removed during surgery (i.e., microscopic *foci*) quickly develop into a new tumor due to their elevated aggressiveness and resistance to conventional anticancer treatments. In this scenario, intensive research activity is focused on the realization and testing of innovative drugs and nanomedicines able to successfully counteract the proliferation of GBM cells.^5^ To be effective and safe, CNS anticancer drugs must efficiently cross the blood–brain barrier (BBB) and selectively target the cancer cells, avoiding significant side effects on healthy cells (e.g., neurons, endothelial cells, and astrocytes).

The development of personalized anticancer compounds and nanocarriers is accompanied by the increased need for highly predictive brain cancer models to test their function.^6^ The vast majority of drug development fails during *in vivo* preclinical and clinical testing.^7^ The obtainment of realistic *in vitro* brain tumor-on-a-chip platforms is crucial to accurately select potentially valid candidate compounds in early investigation stages and, consecutively, to attenuate costs and ethical issues of unsuccessful advanced preclinical studies. Reliable brain tumor-on-a-chip platforms should incorporate real-scale 3D fluidic microcapillaries for BBB modeling and biomimetic multicellular cancer models including malignant and nonmalignant cells to investigate both anticancer efficacy and potential side effects following BBB crossing.^6^ Such a device for biomedical research laboratories and pharmaceutical companies, allowing for a straightforward assembling of different cell types into an *in vivo*-mimicking 3D co-culture system, is indeed still missing in the market and in the literature.

Concerning the straightforward assembly of 3D cell cultures, a recent patent of our group describes the automatic and tunable magnetic docking of cell microscaffolds, envisioning the microfabrication of superparamagnetic and/or ferromagnetic scaffolds that can be used for the development of complex multicellular 3D cultures.^8^ The shape and magnetic properties of each scaffold can be modulated to promote self-assembly in a specific/optimal docking configuration (for instance, with co-cultures aligned, assembled by interlocking, etc.). In addition, scaffolds can be exposed to an auxiliary external magnetic field to remotely tune/guide the process.

**FIG. 1:**
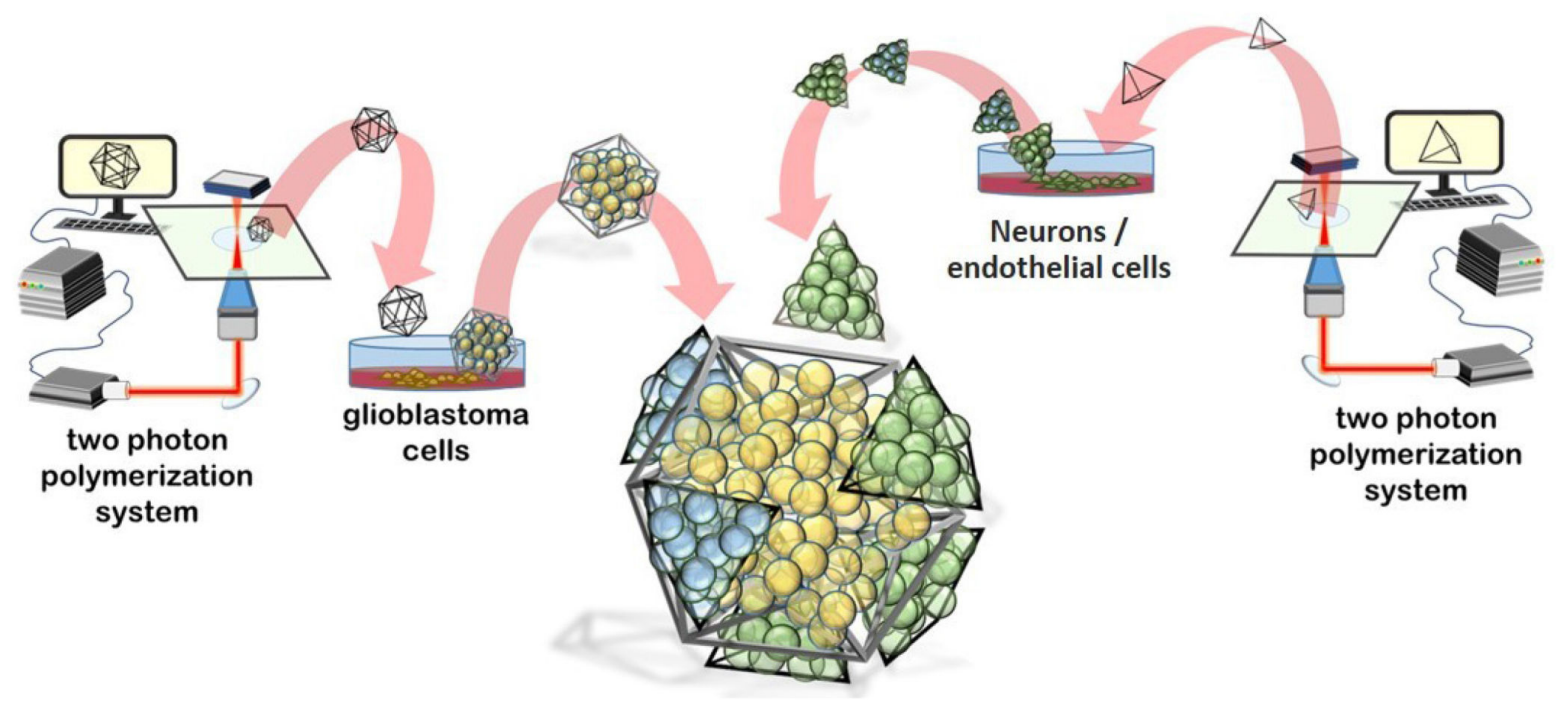
Schematic representation of the self-assembled multicellular 3D system comprising glioblastoma multiforme (GBM) cells and nonmalignant cells (neurons and endothelial cells) in great dodecahedrons (GDs) and tetrahedrons (Ts), respectively.

In this work, we present an innovative multicellular brain tumor-on-a-chip model mimicking the GBM microscopic *foci* interfaced to healthy brain cells [i.e., human neural stem-cell (hNSC)-derived neurons and HCMEC/D human brain endothelial cells]. The magnetic self-assembly of the 3D components of the multicellular system was obtained following the patented method^8^ as described in the schematic representation of Fig. 1. The multicellular 3D system was then integrated into a real-scale microcapillary fluidic model of the BBB. As a proof of concept, nutlin-3a (nut-3a) was screened with our platform in terms of BBB crossing capabilities, apoptotic efficacy against 3D GBM cultures, and side effects on nonmalignant brain cells.

## Results and Discussion

The microscaffolds used for the assembly of the multicellular system were fabricated by two-photon lithography (TPL), an advanced microfabrication approach exploiting the two-photon polymerization (2pp) of dedicated resists for the rapid and precise prototyping of real-3D structures with elevated resolution (i.e., voxel size < 300 nm).^9^ The microscaffolds used for the tumor core is a great dodecahedron (GD), a Kepler–Poinsot solid characterized by 20 small tetrahedron-shaped indentations, which represent the main docking stations of the assembly system. The microscaffolds used for culturing the healthy cell types are small tetrahedrons (Ts).

**FIG. 2:**
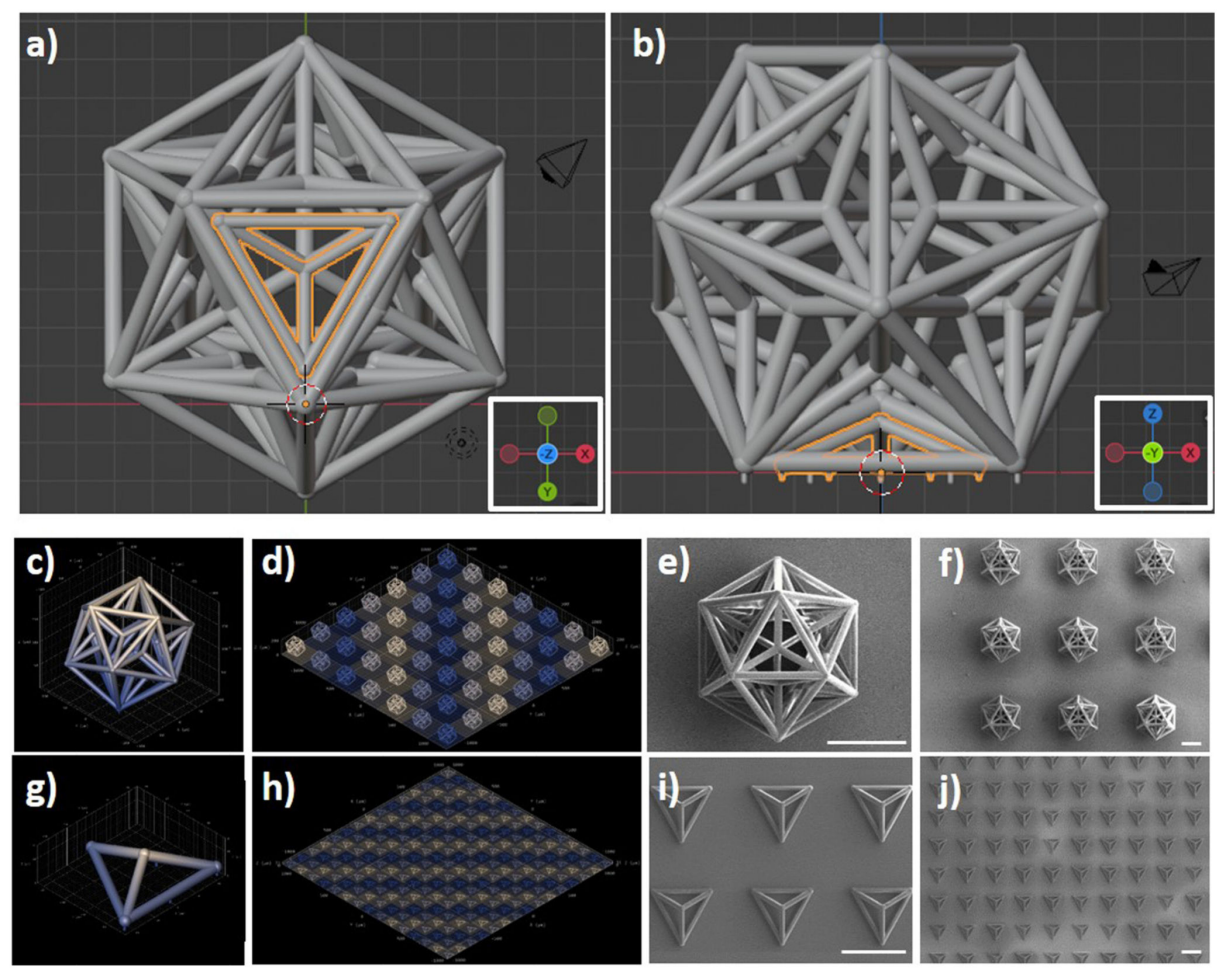
Two-photon lithography (TPL) of the microscaffolds used for the assembly of the multicellular system. (a) Bottom and (b) side view of the 3D rendering of a T into a GD pocket. 3D rendering of (c) a single GD and (d) a matrix of GDs. (e) High magnification and (f) low magnification SEM imaging of the GDs. 3D rendering of (g) a single T and (h) a matrix of Ts. (i) High magnification and (j) low magnification SEM imaging of the Ts. Scale bars 100 *μ*m.

Figures 2(a) and 2(b), respectively, show the 3D rendering (bottom and side views) of a T into a GD pocket. Pillars at the base of the GDs and Ts were designed to reduce the adhesion surface of the scaffolds to the substrate and, therefore, to facilitate their subsequent detachment. The single GDs have a size of 209.4 × 239.5 × 200.0 *μ*m^3^ (*x* × *y* × *z* bounding box) and were fabricated in a 7 × 7 matrix. The Ts have a size of 96.3 × 84.5 × 30.7 *μ*m^3^ (*x* × *y* × *z* bounding box) and were fabricated in a 20 × 20 matrix (Fig. S1 in supplementary material). A representative microscope time-lapse imaging of the TPL of a GD is reported in the supplementary material (Fig. S2 and video S1). A Ti–Ni–Ti triple-magnetic layer was deposited on the fabricated structures: a 60 nm Ti layer was deposited as a primer for a second 60 nm Ni layer, which in turn provided ferromagnetic properties to the scaffold as previously shown with magnetic helical micro-swimmers;^10^ finally, a third 60 nm Ti layer was deposited on Ni for providing biocompatibility. The 3D renderings of a single GD and the matrix of GDs are shown in Figs. 2(c) and 2(d), respectively. SEM imaging of the GDs is shown in Fig. 2(e) (high magnification) and Fig. 2(f) (low magnification). The 3D renderings of a single T and a matrix of Ts are, respectively, shown in Figs. 2(g) and 2(h), while representative SEM scans of fabricated Ts are shown in Figs. 2(i) (high magnification) and 2(j) (low magnification). The high level of reproducibility of the structures can be appreciated by SEM imaging, especially referring to the matrix of the microscaffolds. Moreover, to get an immediate and quantitative hint of the high reproducibility of the procedure, we can consider the smaller features of the structures, i.e., the thickness of the prismatic edges, which show a very low standard deviation among different scaffolds (0.6 *μ*m), corresponding to ˜ 5% of the average thickness (11.5 *μ*m).

**FIG. 3:**
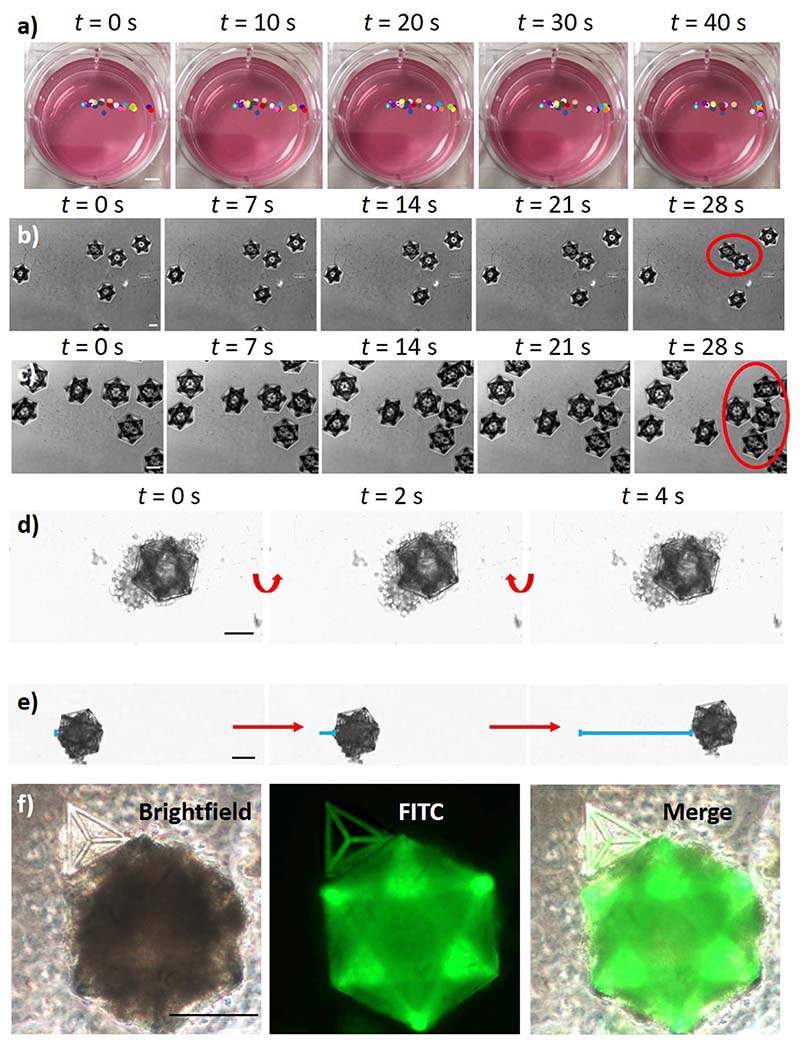
Microscaffold magnetic responsiveness. (a) Time-lapse imaging and tracking of single GDs (colored circles) in response to an external magnet positioned outside of the well on the right (scalebar: 4 mm). Time-lapse images showing the magnetic assembly of the GDs (b) without and (c) in the presence of an external magnet. Once magnetic structures interface with each other, they remain connected and move together (red circles). (d) Rotation and (e) translation of the GDs bearing GBM cells (U87 cells) in response to the external magnet. (f) Magnetic assembly of a GBM cell-bearing GD with brain endothelial cells (hCMEC/D3) on Ts without using an external magnet. Scale bars: in (a) 3.5 mm; in (b)–(f) 100 *μ*m.

The fabricated structures were subsequently detached from the substrates to test their magnetic responsiveness (Fig. 3). The safe detachment of intact structures was achieved by using a tip sonicator (8 W for 150 s; Mini 20 Bandelin Sonopuls) or, alternatively, by gently pipetting with a 1 ml micropipette (Fig. S3 in the supplementary material).

Figure 3(a) shows a time-lapse imaging and tracking of single GDs in a complete cell medium after positioning a Ni–Cu–Ni plated NdFeB magnet (50 × 15 × 15 mm^3^; 324 N attraction force; Supermagnete) outside of the well (on the right). All GDs located at a distance of *l* < 2.5 cm responded to the external magnet moving in its direction. GDs at *l* < 1.3 cm reached the edges of the well in *t* < 20 s, demonstrating satisfactory responsiveness to external magnetic forces. The magnetic assembly of the GDs without and in the presence of an external magnet is reported in the time-lapse images of Figs. 3(b) and 3(c), respectively. The presence of an external magnet induces the movement of the 3D structures, enhancing the probability of their contact and assembly. Once magnetic structures interface with each other, they remain connected and move together. Furthermore, we tested the magnetic responsiveness of the microscaffolds in the presence of cells on the scaffolds [Figs. 3(d)-3(f)]. Specifically, rotation and translation of the GDs bearing GBM cells (U87 cells) are shown in the time-lapse imaging of Figs. 3(d) and 3(e), respectively. In Fig. 3(f), the magnetic assembly of a GBM cell-bearing GD with a brain endothelial cells (hCMEC/D3)-carrying T is shown. Overall, the magnetic tests indicated as the 3D structures can respond to external magnets positioned within a 2.5 cm distance and are prone to their assembly, both in the absence and presence of cells. Furthermore, we demonstrated that with this approach it is possible to tune 3D culture size by both changing the scaffold size (Fig. S4) or by changing the number of cells seeded in the scaffold (Fig. S5). In Fig. S4, GDs with different sizes (104.70 × 119.75 × 100.00 *μ*m^3^, 139.60 × 157.00 × 150.00 *μ*m^3^, and 209.40 × 239.50 × 200.00 *μ*m^3^; *x* × *y* × *z* bounding box) were designed [Fig. S4(a)], fabricated [Fig. S4(b)], and seeded with 50 × 10^3^ cells/cm^2^ [Fig. S4(c)]. In Fig. S5, 30 × 10^3^ and 300 × 10^3^ cells/cm^2^ U87 cell densities were tested on 209.40 × 239.50 × 200.00 *μ*m^3^ scaffolds. Progressively bigger-size tumors were obtained with these two strategies, thereby supporting the versatility and reproducibility of this culture method. Moreover, this approach can be used to obtain the superassembly of multiple 3D cultures of the same cell type to further increase their size (Fig. S6).

**FIG. 4:**
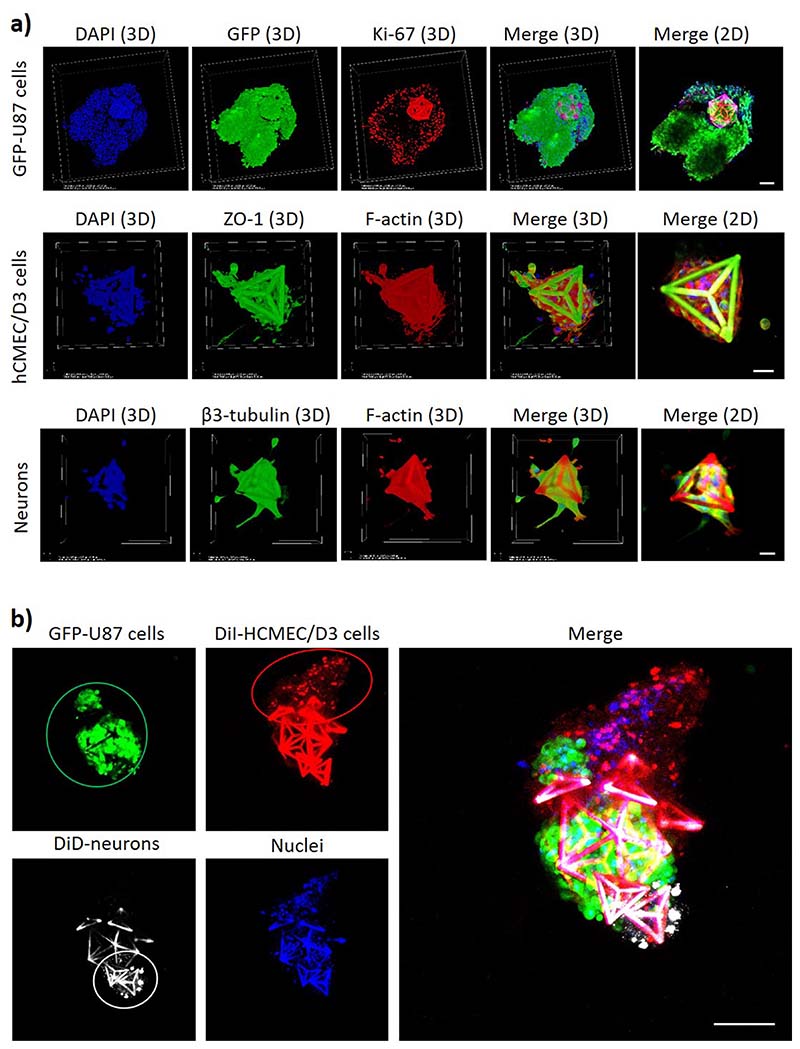
(a) Expression of the Ki-67 proliferation marker in GFP-expressing U87 cells on GDs (top: Ki-67 in red, GFP in green, nuclei in blue; scale bar 100 *μ*m); ZO-1 expression in hCMEC/D3 endothelial cells on Ts (middle: ZO-1 in green, F-actin in red, nuclei in blue; scale bar 20 *μ*m); *β*3-tubulin expression in hNSCs-differentiated neurons on Ts (bottom: *β*3-tubulin in green, F-actin in red, nuclei in blue; scale bar 20 *μ*m). (b) Magnetic self-assembly of a multicellular GBM niche model composed of GD with GFP-U87 GBM cells (green), DiI-stained hCMEC/D3 cells (red) on Ts, and DiD-stained hNSC-derived neurons (white) on Ts. Nuclei of all cell types are shown in blue; scale bar 100 *μ*m.

The magnetic self-assembly of a multicellular co-culture system with GBM cells in a GD and with endothelial and neural cells in Ts is shown in Fig. 4. Before performing the 3D co-culture assembly, the single cultures on microscaffolds were analyzed for the expression of typical molecular markers by immunocytochemistry [Fig. 4(a)]. The expression of the Ki-67 proliferation marker^11^ in the GFP-expressing U87 cells (GFP-U87) shows the proliferative state of the GBM cancer cells cultured on the GDs. Also, the *zonula occludens*-1 (ZO-1) marker of the tight junctions^12^ and the *β*3-tubulin neuronal marker^13^ were, respectively, expressed by hCMEC/D3 endothelial cells and by hNSCs-differentiated neurons on Ts. Concerning hCMEC/D3 endothelial cells in Ts, we would like to highlight that the scope of this work is not obtaining the formation of a vessel or a capillary-shaped structure in Ts, but rather the endothelial cells in Ts represent the tumor-associated endothelial cells recruited by the GBM before the vessel formation. After demonstrating the expression of typical molecular markers of cultured cells, the assembly of the multicellular system mimicking GBM *foci* interfaced to nonmalignant cells of the peritumoral niche was carried out by combining a GFP-U87 GBM cell-bearing GD with Ts seeded with DiI-stained hCMEC/D3 cells and DiD-stained hNSC-derived neurons. The cultures were deposited with a micropipette in the same well and left for 24 h in the presence of the external magnet under the well. At 24 h, nuclei were stained with DAPI, and the 3D multicellular co-culture was imaged with a confocal laser scanning microscope [CLSM; C2s system, Nikon; Fig. 4(b)]. The imaging reveals that the GBM cells (in green) are interfaced with the nonmalignant cell types (brain endothelial cells in red and neurons in white; nuclei in blue). Interestingly, it is possible to observe a small group of GFP-U87 GBM cells that are disconnected from the main colony in the GD, and interfaced with the hCMEC/D3 cells. This could be attributed to GBM cell migration phenomena: in this regard, recent investigations with co-cultures of GBM spheroids and endothelial cells in collagen gels suggested that endothelial cells promote the migration of GBM cells.^14^ Although such investigations are out of the scope of this work, future studies with our model may be directed to explore and inhibit the molecular mechanisms of the GBM invasion in the peritumoral zone.

**FIG. 5:**
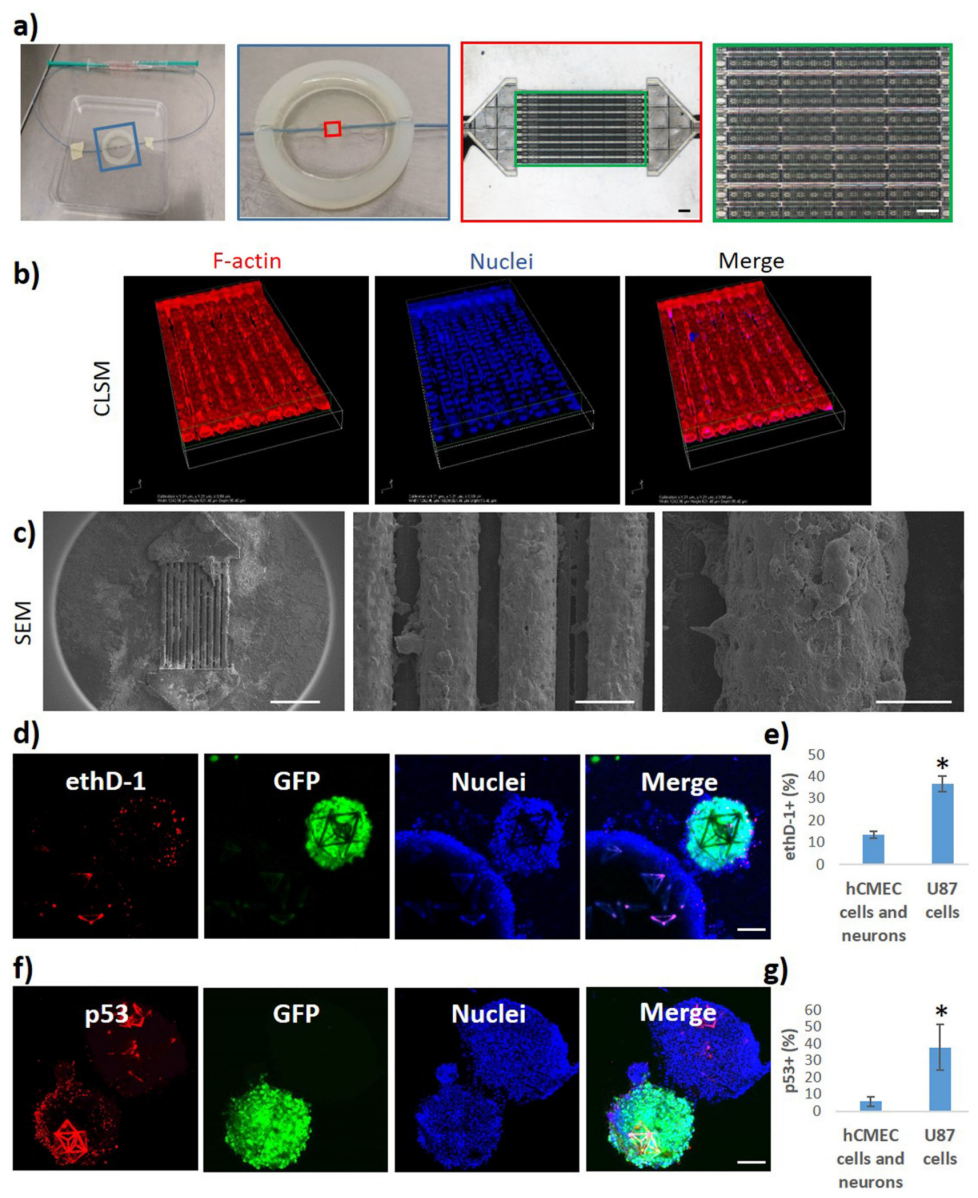
The multicellular GBM niche model was used in combination with a real-scale 3D microfluidic BBB system. (a) Photographs (left) and microscope images in brightfield (right) of the fluidic system with porous microtubes mimicking brain microcapillaries before endothelial cell seeding. (b) Confocal imaging and (c) SEM scans of the hCMEC/D3 endothelial cells surrounding the microtubes. (d)–(g) Anticancer effects of the BBB-crossed nut-3a (5.1 ± 60.6 *μ*M) on the GBM niche model (24 h treatment): (d) confocal imaging and (e) bar graph showing the viability of malignant cells (GFP-U87 GBM cells) in GDs and the viability of nonmalignant cells (hCMEC/D3 brain endothelial cells and hNSC-derived neurons) in Ts in terms of cells positive for ethD-1; (f) confocal imaging and (g) bar graph showing the p53 expression in GFP-U87 GBM cells in GDs and in nonmalignant cells in Ts. * *p* < 0.05%. Scale bars: in (a) 100 *μ*m; in (c) 500, 50, and 20 *μ*m (from left to right); in (d) and (f) 100 *μ*m.

The multicellular GBM niche model was used in combination with a previously described real-scale 3D BBB system^15,16^ to test the anticancer efficacy of BBB-crossed drugs (Fig. 5).

Briefly, the BBB model is a fluidic system with porous microtubes mimicking brain microcapillaries [Fig. 5(a)]. The hCMEC/D3 endothelial cells seeded on the scaffolds surround the microtubes as shown by confocal [Fig. 5(b)] and scanning electron microscopy [SEM; Fig. 5(c)] imaging. The nutlin-3a (nut-3a) anticancer drug was selected as testing molecule, since previous evidence indicated selective anticancer effects toward GBM cells.^17,18^ Nut-3a, an inhibitor of the murine double minute 2 (MDM2), is an anti-proliferation and pro-apoptotic drug that induce the stabilization and activation of the apoptotic p53 pathway in malignant cells having DNA mutations/damages and the wild-type form of p53 (e.g., U87 cells but not p53-mutated T98G cell line).^19^ Preliminary WST-1 viability tests on 2D cultures showed that 5 *μ*m nut-3a significantly decreases the viability of GFP-U87 GBM cells after 24 h (70.2 ± 1.1%; *p* < 0.05; ANOVA followed by HSD *post hoc* test) but does not significantly affect the viability of hCMEC/D3 brain endothelial cells (95.6 ± 11.0%; *p* < 0.05; ANOVA followed by HSD *post hoc* test) and the viability of hNSC-derived neurons (86.7 ± 1.7%; *p* < 0.05; ANOVA followed by HSD *post hoc* test), thereby demonstrating selective anticancer efficacy (Fig. S7). Lower nut-3a concentrations (1 and 2 *μ*m; 24 h incubation) were not able to significantly affect the viability of all three cell types, while higher concentrations (20 *μ*m; 24 h incubation) significantly reduced the viability of all the cell types, probably because of aspecific cytotoxicity. The crossing of the nut-3a from the intratubular to the extratubular space of the fluidic 3D BBB model has been measured by high-performance liquid chromatography (HPLC; results reported in the supplementary material in Fig. S8). A fluid flow of 1 mm/s was imposed in the micro-capillaries, and nut-3a 300 *μ*m was pumped into the system, while the extratubular nut-3a concentration was measured at *t* = 10 min (0.96 ± 0.42 *μ*m), *t* = 90 min (5.12 ± 0.6 *μ*m), and *t* = 240 min (9.97 ± 0.8 *μ*m).

Since 5 *μ*m nut-3a showed selective anticancer efficacy in 2D cultures (Fig. S7) and the crossing of nut-3a through the BBB model was comparable at *t* = 90 min (5.12 ± 0.6 *μ*m, Fig. S8), the following experiment combining the BBB model and 3D multicellular tumor niche systems was performed by pumping 300 *μ*m nut-3a for *t* = 90 min. At *t* = 90 min, the fluidic pump was switched off, and the syringe containing the cell medium with the drug was substituted with a syringe with plain medium. The co-culture was left for 24 h in the incubator before assessing the viability of malignant cells (GFP-U87 GBM cells) in GDs and the viability of nonmalignant cells (hCMEC/D3 brain endothelial cells and hNSC-derived neurons) in Ts in terms of cells positive for ethidium homodimer-1 [ethD-1, Fig. 5(d)]. The results showed a significantly higher % of ethD-1^þ^ malignant cells (36.7% ± 3.6%) *vs* nonmalignant cells [13.5% ± 1.5%; Fig. 5(e)]. Immunofluorescence analyses were then performed to investigate whether the increased cell death was specifically induced by nut-3a through the activation of the p53 apoptotic pathway [Fig. 5(f)]. Our findings report a significantly higher % of p53^þ^ malignant cells (37.8% ± 13.4%) *vs* nonmalignant cells [5.8% ± 2.9%, Fig. 5(g)], suggesting that the higher % of GBM dead cells is associated with a higher expression of p53, which is known to be pharmacologically activated by nut-3a.^17^ Overall, our results confirmed nut-3a to be a promising brain drug, inducing selective anticancer effects on GBM cells when delivered in specific concentration ranges. Nut-3a maintained functional anticancer activity following BBB crossing. However, the administration of this drug must be carefully dosed to remain within the operative range of functional selectivity.

## Methods

### Design and microfabrication

The scaffolds and the microfluidic chips have been fabricated on glass substrates coated with an indium tin oxide (ITO) nanometric layer and suitable for two-photon lithography (TPL) using a negative tone IP-S photoresist (Nanoscribe GmbH) with a Photonic Professional system (Nanoscribe GmbH) equipped with a laser beam centered at a wavelength of 780 nm (Calman laser source). The substrates were previously rinsed with acetone, isopropyl alcohol (IPA), and de-ionized water. A drop of IP-S photoresist was cast on the glass, and the objective (25×, NA 0.8) of the instrument was put in immersion in the photoresist.

Great dodecahedrons (GDs) and tetrahedrons (Ts) were designed in Blender, with cylindrical sides of length 120 *μ*m and diameter 10 *μ*m, and of length 95 *μ*m and diameter 6.5 *μ*m, respectively, to ensure the interconnection. The structures were supported on the glass slide using three 5 *μ*m-thick cylindrical pillars designed at the base of the cages. This solution was adopted to promote the detachment of the structures from the substrate when desired. Both the dodecahedral and the tetrahedral cages were fabricated with a hatching distance of 0.5 *μ*m, slicing distance of 1 *μ*m, writing speed of 10 mm s^−1^, and laser power of 20 mW. Finally, they were developed for 15 min in propylene glycol methyl ether acetate (PGMEA, Sigma-Aldrich) and rinsed with IPA and de-ionized water for 5 min. The Ti–Ni–Ti triple sputtering deposition of the samples was then performed by using an RF/DC magnetron sputtering system from Kenosistec Srl. Specifically, Ti sputtering deposition was carried out at 90 W for 4 min both for the first and last layer. Ni sputtering deposition was performed at 80 W for 8 min. All the processes have been performed at 7 × 10^−2^ mbar in Ar. Finally, GDs and Ts were removed by gently pipetting or by using a tip sonicator (8 W for 150 s; Mini 20 Bandelin Sonopuls) in Dulbecco’s phosphate-buffered saline (DPBS) and separately transferred into non-adherent 24 wells for cell culturing.

The microfluidic chip was fabricated following the printing parameters adopted in a previous work of our group.^16^ Briefly, the IP-S photoresist was selectively exposed to different doses of radiation for the different components of the microfluidic system. The capillaries were fabricated with a writing speed of 20 mm s^−1^ and a laser power of 18 mW, the joints with a writing speed of 20 mm s^−1^ and a laser power of 20 mW, while the rest of the device with a writing speed of 100 mm s^−1^ and a laser power of 50 mW. Hatching distance and slicing distance were 0.5 and 1 *μ*m, respectively. The sample was then developed for 45 min in PGMEA and rinsed with IPA and de-ionized water for 10 min. Finally, two polyether ether ketone (PEEK) tubes (1/32″ o.d. 0.010″ i.d.) of a length of 20 cm were glued to the connectors using a UV curable resin (AA 3494 Loctite).

### Cell cultures

The human cerebral microvascular endothelial cell line hCMEC/D3 (Merck Millipore) was selected as a model of brain endothelial cells due to their large use in central nervous system (CNS) *in vitro* modeling.^20^ hCMEC/D3 cells were cultured in T75 tissue culture flasks using EndoGRO-MV (Sigma-Aldrich), supplemented with EndoGRO-MV Supplement kit (5% heat-inactivated FBS, 5% L-glutamine, 0.2% EndoGRO-LS supplement, 5 ng ml^−1^ rhEGF, 1 *μ*g ml^−1^ hydrocortisone hemisuccinate, 0.75 U ml^−1^ heparin sulfate, 50 *μ*g ml^−1^ ascorbic acid; Sigma-Aldrich), and 1% penicillin–streptomycin (100 IU ml^−1^ of penicillin and 100 *μ*g/ml of streptomycin; Gibco) as previously described.^16^ U87 cells (ATCC HTB-14™) and GFP-expressing U87 cells (Cellomix) were cultured using high-glucose Dulbecco’s modified Eagle medium (DMEM; Sigma-Aldrich) supplemented with 10% heat-inactivated FBS (Gibco), 1% L-glutamine (stock 200 mM; Gibco), 1% sodium pyruvate (stock 100 mM; Gibco), and 1% penicillin–streptomycin (100 IU ml^−1^ of penicillin and 100 *μ*g ml^−1^ of streptomycin; Gibco). For hCMEC/D3 and GFP-expressing U87 cell passaging, the cell culture medium was removed, cells were washed twice with Ca^2+^- and Mg^2+^-free DPBS (Sigma-Aldrich), and incubated for 5 min with trypsin (Sigma-Aldrich) before centrifuging and re-seeding.

The human neural stem cell line (hNSCs; Takara, Y40060) was also used in this work. hNSCs were cultured in RHB-A medium (Takara) containing recombinant human EGF (20 ng ml^−1^; Peprotech) and FGF-2/bFGF (20 ng ml^−1^; Peprotech) in cell culture flasks pre-coated with 10 *μ*g/ml mouse laminin (4 h; Thermo Fisher

Scientific). For hNSC passaging procedures, cell culture medium was removed, cells were washed twice with Ca^2+^- and Mg^2+^-free DPBS (Sigma-Aldrich), and incubated for 5 min in StemPro^™^ Accutase^™^ (Thermo Fisher Scientific) before centrifuging and re-seeding.

For the experiments on GD-size tuning, U87 cells were cultured with their complete medium for 24 h at 50 × 10^3^ cells/cm^2^ in non-adherent conditions in wells containing single GDs of different sizes (104.70 × 119.75 × 100.00 *μ*m^3^, 139.60 × 157.00 × 150.00 *μ*m^3^, and 209.40 × 239.50 × 200.00 *μ*m^3^; *x* × *y* × *z* bounding box). Subsequently, bright field imaging was performed with an optical microscope (Eclipse Ti-E, Nikon). GDs with 209.40 × 239.50 × 200.00 *μ*m^3^ were then selected and used for the following tests. For the experiments on tumor-size tuning, 30 × 10^3^ and 300 × 10^3^ cells/cm^2^ U87 cell densities were tested.

For the superassembly of multiple 3D cultures of the same cell type, U87 cells were cultured with their complete medium for 24 h at 50 × 10^3^ cells/cm^2^ in non-adherent conditions in wells containing single GDs. Then, GDs were collected in the same well and cultured for further 24 h and imaged with epifluorescence microscope (Eclipse Ti-E, Nikon).

For the cell co-culture experiments, hNSCs were seeded at 40 × 10^3^ cells/cm^2^ on laminin pre-coated Ts and treated for 4 days with a first differentiation medium composed of RHB-Basal^™^ medium supplemented with 0.5% NDiff^®^ N2-AF, 0.5% B-27, and 10 ng/ml of recombinant human FGF-basic (Peprotech). The medium was then replaced with a second differentiation medium composed of RHB-Basal and Neurobasal Medium without phenol red (1:1 v/v; Thermo Fisher Scientific), supplemented with 0.25% NDiff N2-AF, 0.5% B-27 supplement, 10 ng/ml bFGF, and 0.5% GlutaMAX (Thermo Fisher Scientific) for other 3 days. At day 4 of hNSC differentiation, the hCMEC/D3 and GFP-U87 cells were seeded on Ts (50 × 10^3^ cells/cm^2^) and GDs (50 × 10^3^ cells/cm^2^), respectively. On day 7 of hNSC differentiation, the different 3D cell cultures were fixed for immunofluorescence analysis or transferred to the same well for co-culturing in non-adherent conditions for 24 h with DMEM (Sigma-Aldrich), supplemented with 10% heat-inactivated FBS (Gibco), 1% L-glutamine (stock 200 mM; Gibco), 1% sodium pyruvate (stock 100 mM; Gibco), and 1% penicillin–streptomycin (100 IU ml^−1^ of penicillin and 100 *μ*g ml^−1^ of streptomycin; Gibco).

### Cell staining

Several staining procedures were followed to visualize the various cells used in this work through confocal microscopy. For co-culture imaging, cells were stained either with Vybrant DiI or DiD dyes (5 *μ*m of DiD for hNSCs and 5 *μ*m of DiI for hCMEC/D3; Thermo Fisher Scientific). Briefly, cells were incubated in full media containing either Vybrant DiI or DiD for 30 min at 37 °C. After the incubation with the dye, cultures were washed twice through centrifugation in DPBS and then transferred to the same well for cell co-culturing. Cell nuclei were stained through incubation with DPBS containing Hoechst 33342 (5 *μ*g ml^−1^ for 30 min; Invitrogen) just before imaging.

For immunostaining procedures, cells were fixed in 4% paraformaldehyde (PFA) for 20 min at 4 °C. Fixed cells were then incubated with Triton X-100 (1:1000 dilution in PBS) for 20 min at RT followed by a 40 min incubation in a blocking solution (10% goat serum in PBS). Cells were then incubated for 90 min at RT with a solution of 10% goat serum in PBS containing the primary antibody. Specifically, GFP-U87 cells were incubated with anti-Ki-67 antibody produced in rabbit (1:150 dilution; Millipore), HCMEC/D3 cells were treated with primary rabbit antibody anti-ZO-1 (2.5 *μ*g ml^−1^, Abcam), and differentiated hNSCs were incubated with anti-tubulin *β*-III antibody produced in rabbit (0.3 *μ*g ml^−1^; Sigma-Aldrich). After incubation with the primary antibody, differentiated hNSCs and HCMEC/D3 cells were washed twice with PBS containing 10% goat serum and then incubated for 1 h in a solution containing 5 *μ*g ml^−1^ of Hoechst 33342 (Invitrogen), 10 *μ*g/ml of F(ab')2-goat anti-Rabbit IgG (H + L) Alexa Fluor 488 conjugate (Invitrogen), and 2.5 *μ*g ml^−1^ of TRITC-phalloidin (Sigma) for 1 h at 37 °C. GFP-U87 cells were treated with a solution of 10% goat serum in PBS containing 5 *μ*g ml^−1^ of Hoechst 33342 (Invitrogen) and a TRITC anti-rabbit secondary antibody (1:250, Invitrogen) for 1 h at 37 °C. After three PBS rinsing steps, images were acquired by a confocal microscope (C2s, Nikon).

### Metabolic activity assay

To assess the effects of nutlin-3a (nut-3a) on the viability of the different cultures, the WST-1 assay (2-(4-iodophenyl)-3-(4-nitophenyl)-5-(2,4disulfophenyl)-2H-tetrazoilium monosodium salt; Biovision) was used. Briefly, U87-GFP, hCMEC/D3, and hNSCs cells were separately seeded on adherent 24-well plates at 25 × 10^3^ cells/cm^2^. hNSCs were seeded on laminin-coated wells and differentiated into neurons as previously described before exposure to nut-3a. Cells were incubated with their culture media (or differentiation media in the case of hNSCs cells) with nut-3a at various concentrations (1, 2, 5, and 20 *μ*m) or only DMSO (the vehicle, 1:1000 dilution) for 24 h. Thereafter, cell metabolic activity was assessed through the WST-1 assay as previously described.^21^

### Nut-3a BBB crossing and tumor niche treatment

The biohybrid device was seeded with hCMEC/D3 (13 × 10^4^ cells/cm^2^). On day 5, the BBB model was connected to the pumping system filled with cell culture medium supplemented with 300 *μ*m of Nut-3a, and a 1 mm/s intratubular flow was imposed.^15^ The extratubular crossing of nut-3a through the endothelial layer was quantified at various time points (10, 90, and 240 min) by high-performance liquid chromatography (HPLC) with a Shimadzu LC-20AT, using a C-18 column (150 × 4.6 mm^2^ i.d., 5 *μ*m particle size) similarly as described in a previous work.^22^ Briefly, a 20% H_2_O/80% CH_3_OH mobile phase was pumped at 1 ml/min flow (isocratic modality). The retention time of nut-3a was 4.6 min, and peak intensities were monitored by the detector at 190 nm. At the end of the experiment, samples were fixed and stained for F-actin and nuclei as described earlier. 3D CLSM reconstruction of the BBB model was carried out using a C2s system (Nikon). The assembly of the multicellular system for the tumor niche formation was carried out on day 7 of hNSC differentiation, by combining a GFP-U87 GBM cell-bearing GD with Ts seeded with DiI-stained hCMEC/D3 cells and DiD-stained hNSCs-differentiated neurons. The 3D cultures grown in non-adhesive conditions were placed with a micropipette in the same well and left for 24 h with DMEM (Sigma-Aldrich), supplemented with 10% heat-inactivated FBS (Gibco), 1% L-glutamine (stock 200 mM; Gibco), 1% sodium pyruvate (stock 100 mM; Gibco), and 1% penicillin–streptomycin (100 IU ml^−1^ of penicillin and 100 *μ*g ml^−1^ of streptomycin; Gibco) in the presence of the external magnet under the well. The obtained 3D multicellular cultures with GFP-U87, HCMEC/D3, and differentiated hNS were carefully collected with the micropipette and placed at a 3.5 ± 0.6 mm distance from the BBB system to perform drug testing. The pumping system was filled with nut-3a and activated as described above. At *t* = 90 min, the fluidic pump was switched off, and the syringe containing the cell medium with the drug was changed with a syringe with the plain medium. The co-culture was left for 24 h in the incubator in the presence of the nut-3a that crossed the BBB model. After the microfluidic experiment, 3D multicellular cultures were stained with Hoechst 33342 and ethidium homodimer-1 (2 *μ*m; Thermo Fisher Scientific) for 20 min at 37 °C in cell culture medium, washed twice in DPBS, and imaged with the confocal microscope. To assess the induction of apoptosis caused by nut-3a after BBB crossing, 3D multicellular cultures were fixed in PFA 4% for 20 min at 4 °C and subjected to an immunostaining procedure using a primary mouse anti-p53 antibody (10 *μ*g ml^−1^; Abcam) and a TRITC-conjugated secondary anti-mouse antibody (1:250 dilution; Millipore), as previously described. After the immunostaining, cells were imaged through the confocal laser scanning microscope.

### Scanning electron microscopy imaging (SEM)

For the SEM imaging of the microfluidic system seeded with hCMEC/D3 cells, samples were fixed in PFA as previously mentioned, washed with Milli-Q water, and double-fixed with glutaraldehyde 2.5% in Milli-Q water for 2 h at 4 °C. After the glutaraldehyde fixation procedure, samples were dehydrated with increasing ethanol concentrations (25, 50, 75, and 100% in water, incubation of 5 min for each step), dried, gold-sputtered using an SC7620 Mini Sputter Coater/Glow Discharge System at 20 mA for 30 s, and eventually imaged with a SEM system (Helios NanoLab 600i FIB/SEM, FEI). For the imaging of GDs and Ts, samples were gold-sputtered and imaged as described mentioned earlier.

## Conclusion

The lithography approach adopted in this work exploits the two-photon polymerization phenomenon. The fundamental difference from other lithography and other additive manufacturing is that TPL allows for a real 3D scaffold fabrication, and thanks to the non-linearity of the local polymerization method, it is possible to reach sub-micrometric resolutions. By adopting this fabrication technique, we were able to generate non-degradable 3D prismatic-shaped magnetic scaffolds colonizable by cells. Furthermore, the shape-coding of the scaffolds allowed us to detect the migration phenomena from one structure (GD-shaped) to another one (T-shaped), identified by confocal imaging of their assembly by exploiting the autofluorescence of the microscaffolds. Alternative assembly approaches reported in the literature, such as the ones exploiting magnetic iron oxide (MIO)-loaded hydrogels, conversely do not allow for a real 3D assembly and shape-coding.^23^ Eventually, our approach differs from other magnetic-based co-culture methods that involve the cellular uptake of magnetic nanoparticles or beads,^24^ which are known to interfere with biological processes through different biochemical pathways, thus affecting the experimental outcome.^25^

Our findings show that our brain tumor-on-a-chip system represents an excellent biomimetic platform for testing drug delivery through biological membranes and the functional selectivity of the anticancer effects. The development of a 3D co-culture system recapitulating the GBM microenvironment, in principle, can be exploited not only for performing predictive tests on anticancer drugs, but it may also result in a versatile platform to test other therapeutic approaches (e.g., antiangiogenic treatments) acting on nonmalignant cells (e.g., neuronal/glial progenitors, astrocytes, microglia, and endothelial cells) that support the tumor growth.^26^ The incorporation of the immune cells in the platform will be fundamental for performing preliminary investigations on the efficacy of novel personalized immunotherapy approaches. Moreover, such a multicellular model mimicking the complexity of the GBM niche may represent a valuable tool in glioma development research to reveal the still unclear origins and causes of this tumor (i.e., testing the stem cell theory, the de-differentiation hypothesis, and the midway theory).^27^ Finally, the potential of the magnetic co-culture assembling can be exploited in future investigations to recapitulate the 3D architecture and function of different multicellular units, such as the neuromuscular junction or the islets of Langerhans, in physiologic and pathologic conditions.

## Supplementary Material

SI

Video S1

Video S2

Video S3

Video S4

Video S5

## Data Availability

The data that support the findings of this study are openly available in Zenodo at https://doi.org/10.5281/zenodo.8013889, Ref. 28.
