# Supplementary material for: Magnetic self-assembly of 3D multicellular microscaffolds: A biomimetic brain tumor-on-a-chip for drug delivery and selectivity testing
Source: APL Bioeng. Author manuscript; Available in PMC 2023 Aug 1. (PMC10375466; doi:10.1063/5.0155037)
Supplement: SI [file EMS181843-supplement-SI.DOCX]

SUPPLEMENTARY MATERIAL

**Magnetic self-assembly of 3D multicellular microscaffolds: A biomimetic brain tumor-on-a-chip for drug delivery and selectivity testing**

Attilio Marino,^1,^* Matteo Battaglini^1^, Alessio Carmignani^1,2^, Francesca Pignatelli^1^, Daniele De Pasquale^1^, Omar Tricinci^1^, Gianni Ciofani^1,^*

1. Istituto Italiano di Tecnologia, Smart Bio-Interfaces, Viale Rinaldo Piaggio 34, 56025 Pontedera, Italy

2. Scuola Superiore Sant’Anna, The BioRobotics Institute, Viale Rinaldo Piaggio 34, 56025 Pontedera, Italy

*E-mails: [attilio.marino@iit.it](mailto:attilio.marino@iit.it); [gianni.ciofani@iit.it](mailto:gianni.ciofani@iit.it)


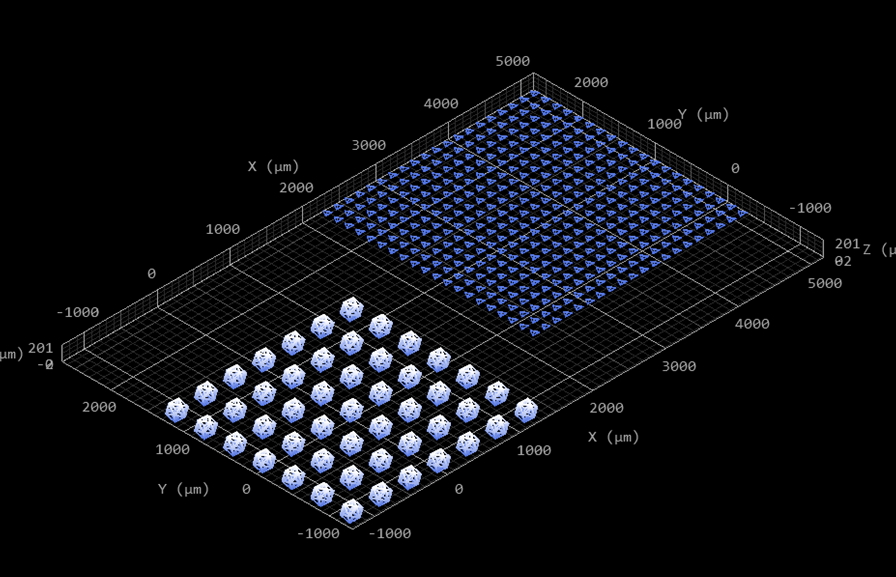


**Figure S1.** Design of a GDs (7 × 7) and a Ts (20 × 20) matrix.


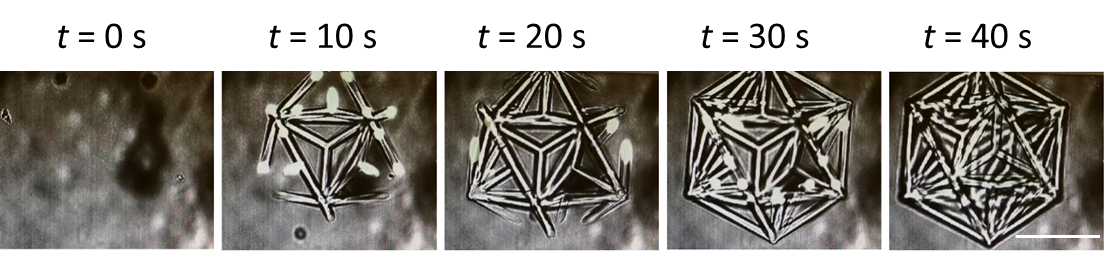


**Figure S2.** Microscope time-lapse imaging of the TPL of a GD. Scale bar 100 μm.


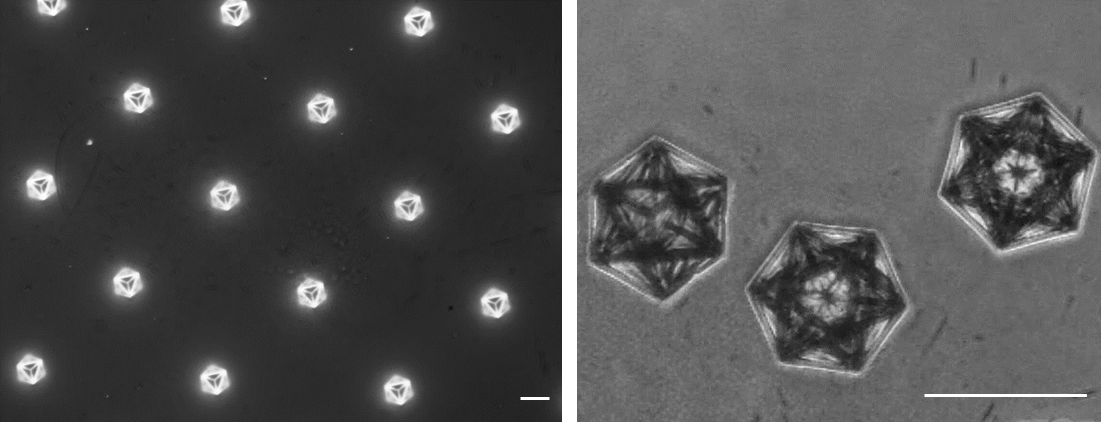


**Figure S3.** Safe detachment of the intact magnetized microscaffolds. Scale bars 200 µm.


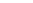

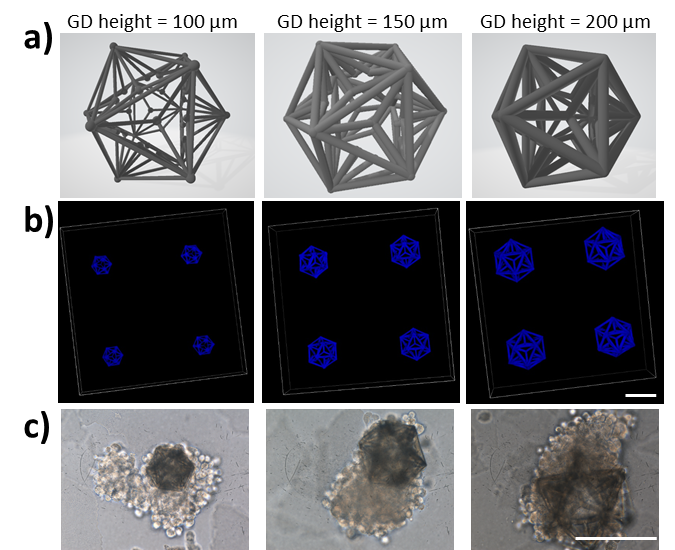


**Figure S4.** Tuning of 3D culture size by scaling the scaffold features. a) design, b) confocal laser scanning imaging of fabricated scaffolds, and c) 3D U87 cell culture in non-adherent conditions on GDs with different size: 104.70 µm × 119.75 µm × 100.00 µm (left), 139.60 µm × 157.00 µm × 150.00 µm (middle), and 209.40 µm × 239.50 µm × 200.00 µm (right). Scale bars: 200 µm.


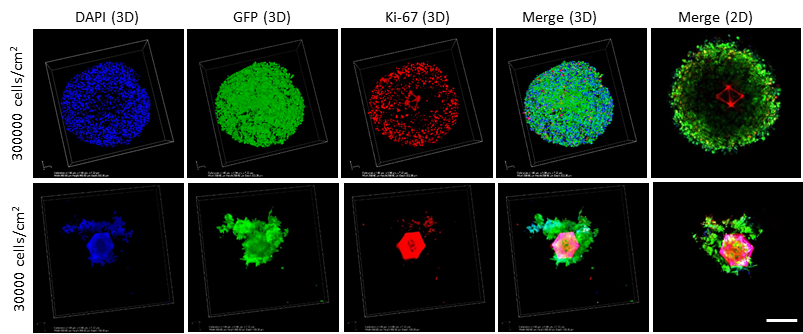


**Figure S5.** Different tumor size obtained by seeding GFP-expressing U87 cells at 30·10^3^ cells/cm^2^ (top) and 300·10^3^ cells/cm^2^ (bottom) on 209.40 µm × 239.50 µm × 200.00 µm scaffolds. 3D confocal reconstruction of nuclei in blue, GFP-U87 cells in green, Ki-67 proliferation marker in red, and merge. The 2D merge of a single stack has been also reported (right). Scale bar: 200 µm.


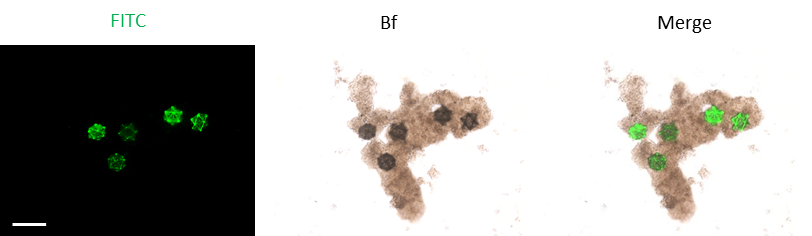


**Figure S6.** Assembly of a large 3D GBM tumor by incubating for 24 h 5 GDs with U87 cells at 50·10^3^ cells/cm^2^. Autofluorescence of the scaffolds (left), bright field (middle), and merged image (right). Scale bar: 400 µm.


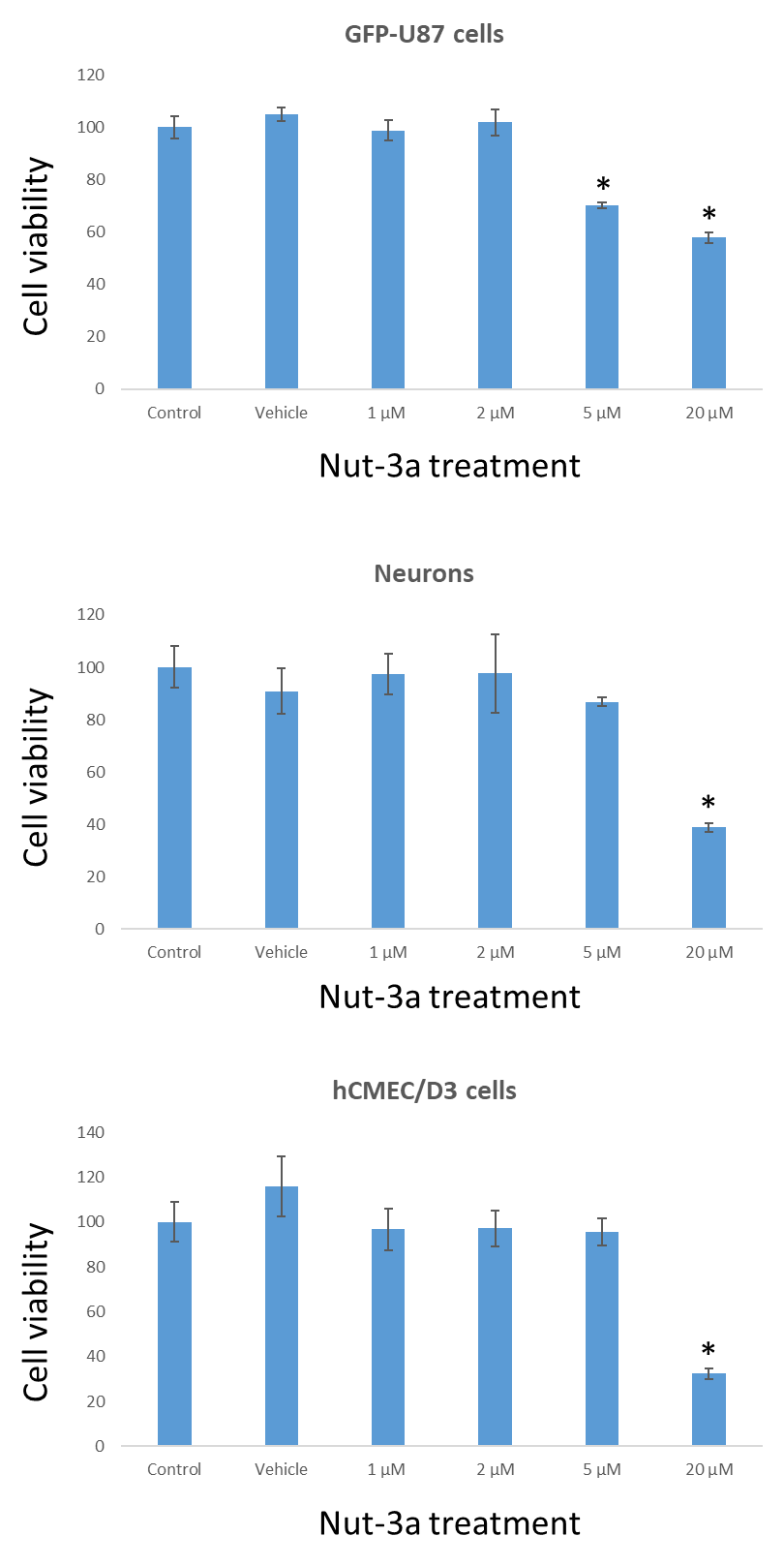


**Figure S7.** WST-1 viability assay on 2D cultures (GFP-U87, neurons, and hCMEC/D3 cells) in response to different nut-3a concentrations (1, 2, 5, 20 µM). Data are normalized as % of control cultures. The vehicle is DMSO (1:1000 dilution in cell medium). * *p* < 0.05%.


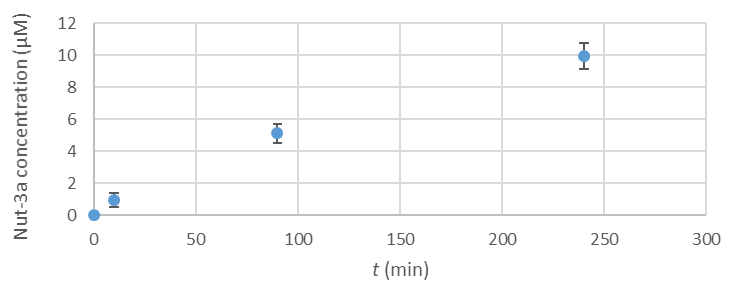


**Figure S8.** Nut-3a concentrations in the extratubular space of the fluidic 3D BBB model were measured by HPLC at *t* = 10 min, *t* = 90 min, and *t* = 240 min from nut-3a administration in the intratubular solution.

**Video S1.** Layer-by-layer two-photon polymerization of a GD.

**Video S2.** Magnetic assembly of the GDs without external magnet.

**Video S3.** Magnetic assembly of the GDs in the presence of an external magnet.

**Video S4.** Rotation of a U87 cells-bearing GD.

**Video S5.** Translation of a U87 cells-bearing GD.
